# Utilization of Rad51C promoter for transcriptional targeting of cancer cells

**DOI:** 10.18632/oncotarget.1792

**Published:** 2014-02-19

**Authors:** Yan Cao, Yan Xu, Lei Zhang, Zhen Li, Ying Jiang, Xiao Tian, Andrei Seluanov, Vera Gorbunova, Zhiyong Mao

**Affiliations:** ^1^ School of Life Sciences and Technology, Tongji University, Shanghai, China; ^2^ Department of Biology, University of Rochester, Rochester, NY, USA

**Keywords:** Rad51C promoter, Rad51 paralogs, cancer therapy, DNA double strand break repair, homologous recombination

## Abstract

Cancer therapy that specifically targets malignant cells with minimal or no toxicity to normal tissue has been a long-standing goal of cancer research. Rad51 expression is elevated in a wide range of cancers and Rad51 promoter has been used to transcriptionally target tumor cells, however, a large size of Rad51 promoter limits its application for gene therapy. To identify novel tumor-specific promoters, we examined expression levels of Rad51 paralogs, Rad51B, Rad51C, and Rad51D as well as Rad52 in a panel of normal and tumor cell lines. We found that Rad51C is significantly overexpressed in cancer cells. The expression was up-regulated by approximately 6-fold at the mRNA level and 9-fold at the protein level. Interestingly, the 2064 bp long Rad51C promoter fragment was approximately 300-fold higher in cancer cells than in normal cells. A construct containing Rad51C promoter driving diphtheria toxin A efficiently killed several types of cancer cells with very mild effect to normal cells. These results underscore the potential of targeting the homologous recombination pathway in cancer cells and provide a proof of principle that the Rad51C promoter fragment can be used to transcriptionally target cancer cells.

## INTRODUCTION

The goal of cancer treatment is selective elimination of cancer cells with minimal effect on normal adjacent tissues or cells. Among numerous gene therapy tools, use of a cancer specific promoter fused with a toxic gene offers unique opportunities for selective targeting of cancer cells. However, only a few promoters were identified with a cancer specific activity. The best-characterized promoter is hTERT[1], which is overexpressed in approximately 90% of tumor types while suppressed in somatic tissues. Tissue specific promoters, such prostate-specific promoters have been used to target prostate cancer cells [2]. Recent research on Rad51, the essential recombinase required for homologous recombination (HR), reveals that the promoter of Rad51 is highly expressed in a panel of cancer cells in comparison to a set of normal cells [3]. Rad51 promoter fused to DTA gene delivered with nanoparticle specifically killed tumors *in vivo* using a xenograft model [4]. Due to genetic and epigenetic diversity of tumors not all the promoters will work well in every type of cancer. Furthermore, tumor-specific promoters such as hTERT, may be expressed in stem and progenitor cells resulting in toxicity of the gene-therapy constructs to non-cancerous cells. Therefore, it is important to expand the list of cancer-specific promoters.

Rad51 is overexpressed in a wide variety of cancer cell types [5, 6]. Consequently, we found that HR repair pathway is elevated in breast cancer cells [7]. Cancer cells may rely on the HR pathway to repair collapsed replication forks generated during uncontrollable replication of the cancer cells. Our group then pioneered the use of Rad51 promoter for cancer gene therapy [3, 4]. However, a limiting factor in the use of Rad51 promoter is its relatively large size 6532bp required to achieve high expression in cancer cells. This size would interfere with efficient packaging into viral vectors further complicating the delivery of the therapeutic construct. In order to identify alternative promoters with high specificity to cancer cells we examined the tumor specificity of Rad51 paralogs Rad51B, Rad51C, Rad51D and Rad52. Rad51 paralogs and Rad52 participate in HR repair alongside Rad51. Rad52 protein facilitates Rad51 nucleoprotein filament formation[8]. The molecular function of the Rad51 paralogs in the HR process is less clear. Loss of any of the Rad51 paralogs sensitizes cells to DNA cross-linking agents and ionizing radiation[9]. In mice, disruption of any Rad51 paralog is embryonic lethal, suggesting an essential role of these proteins in repairing DSBs caused by collapsed DNA replication forks in early embryonic development[10-13]. Biochemically, Rad51 paralogs form two distinct complexes Rad51B-Rad51C-Rad51D-XRCC2 and Rad51C-XRCC3, which possibly play similar roles to BRCA2 by recruiting the major HR recombinase Rad51 to broken ends. Rad51C is the central component of both complexes[9], implying its potentially essential role in HR directed repair. Not limited to its early role in Rad51 recruitment [14], Rad51C has been proposed to facilitate the resolution of Holliday Junction formed at late stage of HR [15, 16]. Furthermore, recent large-scale sequencing studies of breast cancer, ovarian cancer and testicular cancer patients and their families identified Rad51C mutations associated with increased cancer risk [17-21].

Here, we explored the utility of the HR gene Rad51B, Rad51C, Rad51D and Rad52 for transcriptionally targeted therapy of cancer. We found that expression of Rad51C was significantly elevated in the group of cancer cells. Moreover, the expression of firefly luciferase or GFP fused pRad51C was approximately 300 times higher in cancer cells than in normal cells. We then engineered a prototype targeting construct containing a diphtheria toxin A driven by 2064 bp fragment of the Rad51C promoter (pRad51C-DTA). The pRad51C-DTA specifically targeted cancer cells while it had very mild effect on all normal cell lines.

## RESULTS

### Rad51C is highly expressed in cancer cells

**Figure 1:**
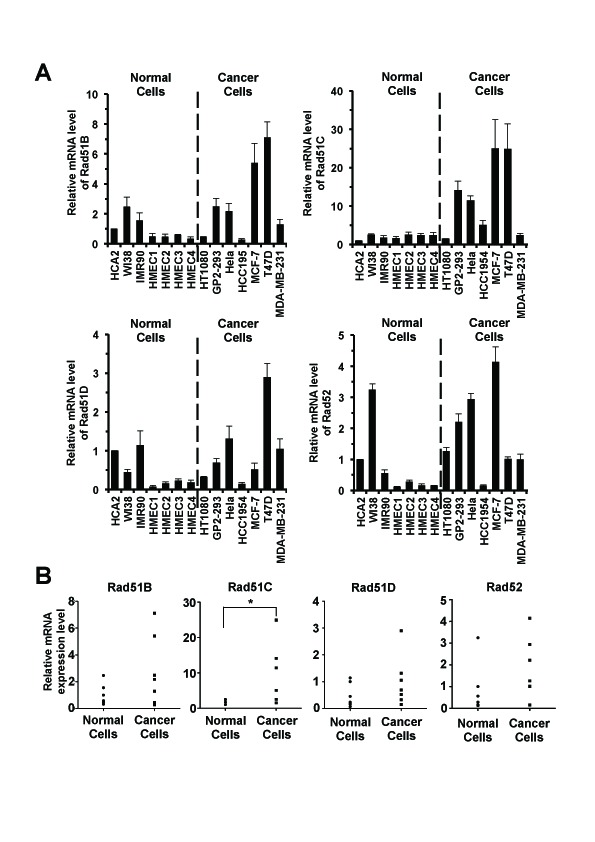
Rad51C transcripts are upregulated in cancer cells A. Quantitative analysis of mRNA expression of Rad51B, Rad51C, Rad51D and Rad52 in normal and cancer cells. Exponentially growing cells were harvested for total RNA extraction. The transcript levels were determined using real time RT-PCR followed by analysis using delta CT method [29]. Then the relative expression level of Rad genes in different types of cells was normalized to that in HCA2 cells. All experiments were repeated at least 9 times. Error bars represent s.d. B. Statistical analysis of expression of Rad51B, Rad51C, Rad51D and Rad52 in normal and cancer cells. Man Whitney U test was employed to examine significance. * The upregulation of Rad51C in cancer cells was statistically significant (*P_MWU_*=0.037).

To identify cancer-specific promoters we first compared the expression levels of Rad51B, Rad51C, Rad51D and Rad52 in a panel of seven normal and seven cancerous cells lines. Normal cells included three lines of normal human fibroblasts HCA2, IMR90 and WI38, and four different lines of normal human mammary epithelial cells HMEC1, HMEC2, HMEC3 and HMEC4. The cancer cell lines included four of breast cancer cells HCC1954, MCF-7, T47D and MDA-MB-231, a fibrosarcoma cell line HT1080, a cervical cancer cell line HeLa and a transformed human kidney cell line GP2-293. We extracted total RNA from the exponentially growing cells, and measured transcript levels by real-time PCR (Figure 1A).

All four Rad genes showed a trend towards higher expression in cancer cells (Figure 1A). Expression of Rad51B, Rad51D and Rad52 was elevated approximately 2-fold compared to normal cells, while the expression of Rad51C was elevated 6-fold. However, only Rad51C result reached statistical significance (Rad51B, *P_MWU_*=0.3176; Rad51D, *P_MWU_*=0.2086; Rad52, *P_MWU_*=0.1282; Rad51C, *P_MWU_*=0.037; Figure 1B). These results make Rad51C promoter a promising candidate for transcriptionally targeted therapy.

### The protein levels of Rad51C are upregulated in cancer cells

**Figure 2:**
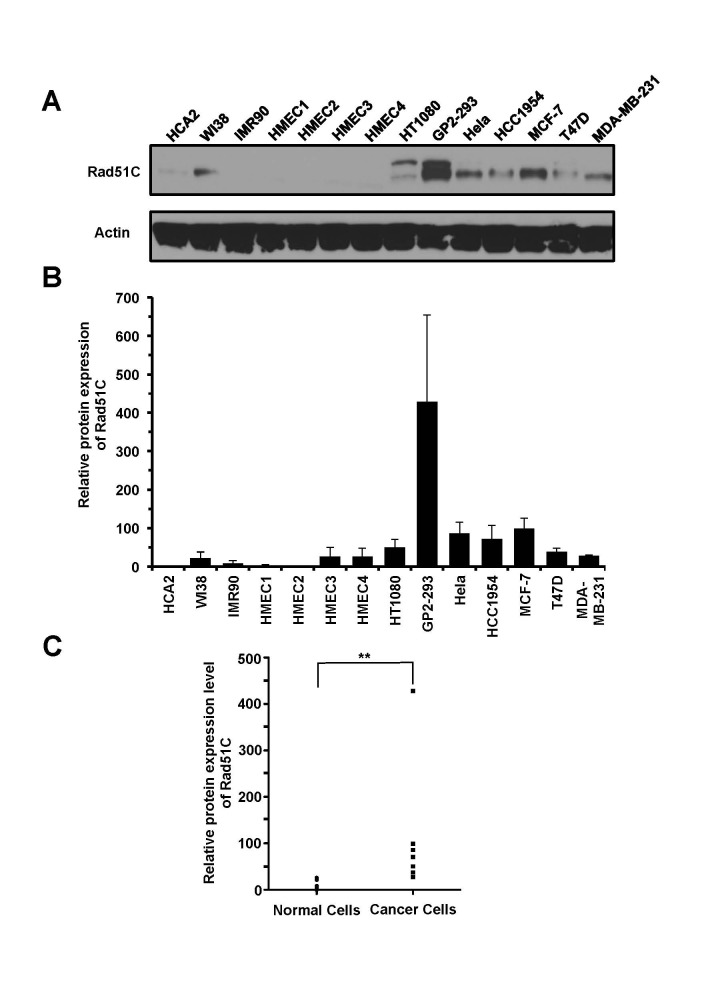
Rad51C protein levels are elevated in cancer cells A. Representative Western blot showing expression levels of Rad51C in normal and cancer cell lines. B. Quantitative analysis of Rad51C protein levels in normal and cancer cells. The Western blot results were quantified with ImageJ software. All experiments were repeated at least three times. Error bars indicate s.d. C. The Man Whitney U test shows that the elevation of Rad51C protein expression in cancer cell lines is statistically significant. ** *P_MWU_*=0.0006.

To further confirm that Rad51C is overexpressed in cancer cells we examined the protein levels by Western blot. The analysis showed that Rad51C protein levels are up-regulated in cancer cells (Figure 2A). On average, Rad51C protein levels were elevated 9-fold (Figure 2B). This increase was statistically significant (*P_MWU_*=0.0006) (Figure 2C).

### The Rad51C promoter fragment is highly active in cancer cells

**Figure 3:**
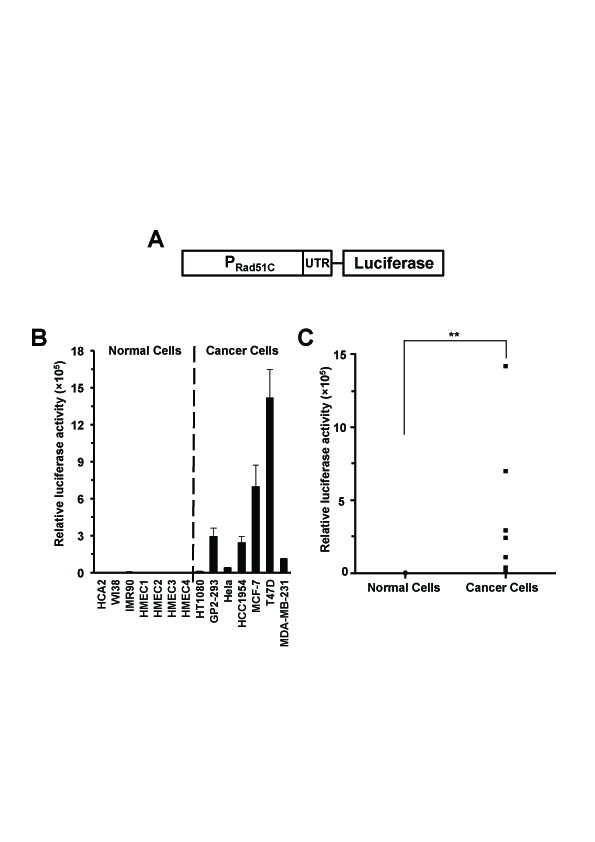
Rad51C promoter is hyperactive in cancer cells A. Diagram of Rad51C reporter construct. B. The activity of Rad51C promoter is strongly enhanced in cancer cells. Cells were transfected with pRad51C-luciferase. The ratio of luciferase activity to GFP+ cells was used as the measure of Rad51C promoter activity. All experiments were repeated more than three times. Error bars indicate s.d. C. The elevation of Rad51C promoter activity was highly significant in cancer cells. ** *P_MWU_*=0.0006.

Since Rad51C showed significant upregulation in cancer cells we proceeded to test whether Rad51C promoter is also hyperactive in cancer cells. We cloned a putative Rad51C promoter fragment, which starts from -1966 upstream to +99 downstream of the transcription start site, into a vector containing firefly luciferase or EGFP gene resulting in a pRad51C-luciferase or pRad51C-EGFP construct. We then tested the promoter activity by transfecting cells with 0.5 μg of pRad51C-luciferase construct or 2 μg pEGFP-N1 as a control for normalizing transfection efficiency. The ratio of luciferase activity versus GFP+ cells was used as a measure of Rad51C promoter activity. Strikingly, pRad51C-luciferase construct was 225-fold more active in cancer cells than in normal cells (*P_MWU_*=0.0006) (Figure 3, Supplementary Figure 1). Similarly, after co-transfecting cells with 0.5 μg of pRad51C-EGFP together with 0.005 μg of pDsRed2-N1 as an internal control for normalizing transfection efficiency, we observed a 343-fold difference of EGFP+ cells/DsRed+ cells between cancer cells and normal cells (*P_MWU_*=0.0006) (Supplementary Figure 2). This differential activity between normal and cancer cells is very promising for future therapeutic applications.

### Rad51C promoter driving expression of Diphtheria Toxin A (DTA) selectively targets cancer cells

**Figure 4:**
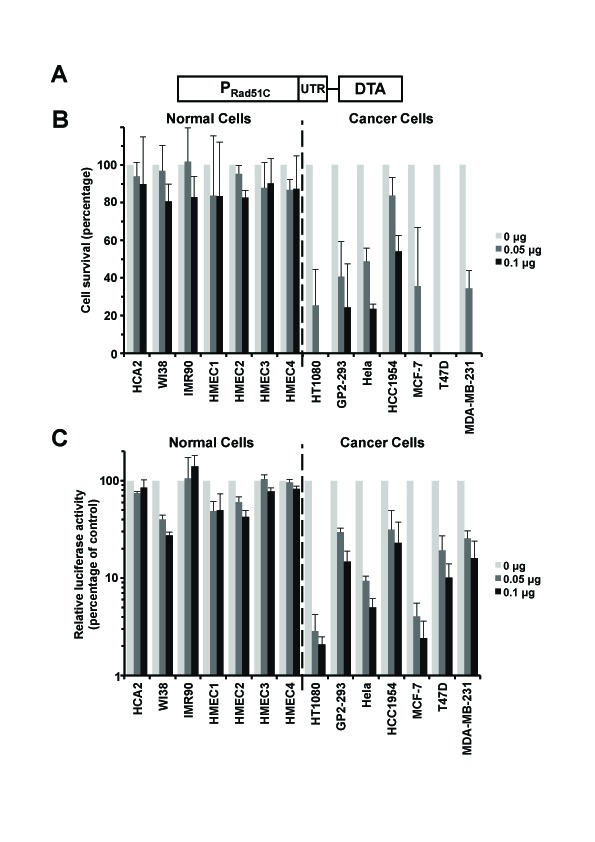
pRad51C-DTA selectively targets cancer cells with minimal toxicity to normal cells A. A diagram of pRad51C-DTA. B. Survival of cells transfected with pRad51C-DTA. Zero, 0.05 μg or 0.1 μg of pRad51C-DTA was transfected into cells using electroporation. At 72 hours post transfection, cells were harvested and cell number was counted on a Millipore Muse machine. The survival rate was calculated as described^16^. All experiments were repeated more than three times. Error bars represent s.d. C. Inhibitory effect on luciferase activity in cancerous cell lines by pRad51C-DTA. Zero, 0.05 μg or 0.1 μg of pRad51C-DTA was cotransfected with 1 μg SV40-luciferase to the 14 cell lines using Fugene. At day 3 post transfection, cells were harvested and luciferase activity was analyzed in cell lysates. All experiments were repeated at least three times. Error bars represent s.d.

We next constructed a prototype therapeutic construct by fusing Rad51C promoter to diphtheria toxin A (DTA) gene generating pRad51C-DTA (Figure 4A). DTA encodes a toxic protein that blocks protein translation in the cell by inactivating eEF2, leading to rapid cell death. We then transfected pRad51C-DTA into the normal and cancer cell lines and counted the number of surviving cells using a Millipore Muse machine. pRad51C-DTA specifically killed all the seven types of cancer cells and had no significant effect on the seven normal cell lines (Figure 4B).

The second approach we used to measure the efficiency of cancer cell killing by pRad51C-DTA was to analyze inhibition of protein synthesis triggered by therapeutic construct. pRad51C-DTA and a plasmid encoding firefly luciferase were co-transfected into the panel of normal and cancer cells, and the level of luciferase activity was compared in the cells transfected with pRad51C-DTA to the control cells that received the luciferase construct only. Protein synthesis measured by the luciferase activity was reduced by ~25% to ~28% in the seven normal cell lines (Figure 4C). In contrast, the inhibitory effect by pRad51C-DTA on the tumor cell lines was much greater, approximately 7-fold and 10-fold with 0.05 μg and 0.1 μg pRad51C-DTA transfected respectively (Figure 4C). In summary these results show a proof of principle that Rad51C promoter can be successfully used to target cancer cells.

## DISCUSSION

We have shown that Rad51C transcript and protein levels are elevated in cancer cells. This is consistent with the previous findings that the levels of partner HR protein Rad51 [5, 6], as well as HR repair efficiency, are up-regulated in cancer cells [7]. Cancer cells may undergo selection for more active HR repair machinery to alleviate replication stress in rapidly dividing cancer cells. Furthermore, active HR may diversify the genomes of cancer cells leading to more rapid loss of heterozygosity and emergence of therapy resistant clones. On the contrary, normal cells with intact cell cycle checkpoints prefer to nonhomologous end joining (NHEJ) rather than HR [22]. Therefore, targeting HR pathway is a promising strategy to selectively kill cancer cells.

Here we have shown that Rad51C promoter is highly active in cancer cells and the pRad51C-DTA construct can target cancer cells with high selectivity. Targeting cancer cells with a construct encoding DTA gene driven by Rad51 promoter had previously shown promise in cultured cancer cells and animal models [3, 4, 23]. Several systems expressing toxic or suicide genes driven by other cancer specific promoters such as hTERT [1], Tyrosinase [24], Survivin [25] and Midkine [26] were developed in the past decade. hTERT is one of the best-characterized tumor-specific promoters. Although hTERT promoter activity is low in normal somatic cells, it is a relatively weak promoter even in cancer cells, which limits its clinical application. The differential in hTERT promoter activity between normal and cancer cells is approximately 10-fold [27]. In contrast, Rad51C promoter activity is on average over 225-fold stronger (with the maximum difference of 3,060-fold) in cancer cells than that in normal cells, which may lead to much higher tumor specificity. This differential between cancer and normal cells is even greater for the Rad51 promoter, when a 6.5 kb promoter fragment is used. However, despite its high selectivity, full length Rad51 promoter is incompatible with the majority of gene delivery tools for gene therapy, such as viral vectors. When Rad51 promoter was truncated to 2 to 3 kb, differential in promoter activity between cancer and normal cells was reduced to 60-fold or less [23]. Importantly, the size of Rad51C promoter required for differential expression in cancer and normal cells is only ~2 kb, which is within the range insert size that can be accommodated by most of viral vectors [28].

The activity of Rad51C promoter fused to a reporter gene was dramatically higher in cancer cells relative to the increase in the endogenous levels of Rad51C transcript. A possible explanation is that the 2 kb Rad51C promoter used in the fusion constructs is missing suppressive regulatory element(s). Alternatively, the endogenous Rad51C transcript may contain binding sites for micro RNAs that are missing in the constructs. Regardless of the cause, this phenomenon could be exploited for targeting cancer cells.

In summary, our study provides evidence that HR pathway is a promising therapeitic target. We demonstrated that Rad51C promoter can be used to transcptionally target cancer cells and identified a 2 kb promoter fragment that provides strong differential expression in cancer cells. This work expands the list of cancer-specific promoters that hold promise for clinical applications.

## MATERIALS AND METHODS

### Cell culture

All cells were cultured in 3% O_2_ and 5% CO_2_ at 37°C in a humidified incubator (Heracell 240i, Thermo Fisher, USA). The growth conditions for the 14 cell lines were as described [3, 7].

### Generation of pRad51C-luciferase, pRad51C-EGFP, and pRad51C-DTA constructs

Rad51C promoter was amplified from genomic DNA isolated from HCA2 cells using primers 5'GCTGAATTCGCATAAGCATGAAATCTCCCT GAAGATAG3' and 5'ACCGGTACCCGCTGCATTTCAAAGCGGAA CGTCTTC3', and sub-cloned into a vector with EcoRI and KpnI. After the promoter sequence was confirmed, we further subcloned Rad51C promoter into pEGFP-N1 by replacing CMV promoter with ApaLI and KpnI, generating pRad51C-EGFP. Then the promoter region was amplified with primers 5'ATTGGTACCGCATAAGCATGAAATCT CCCTGAAGATAG3' and 5'CGCGCTAGCCGCTGCATTTCAAAG CGGAACGTCTTC3', and cloned into pGL3-basic vector to create pRad51C-firefly luciferase construct using KpnI and NotI.

Based on pRad51C-EGFP, we obtained pRad51C-DTA by replacing EGFP ORF with DTA ORF amplified from pROSA26KPN using primers 5'GGCGGTACCGCCACCATGGATCCTG ATGATGTTGTTGATTCTTC3' and 5'GTCGCGGCCGCTTAGAGCTTTAAATCTCTG3'.

### Real-time PCR

Total RNA was extracted from cells that were seeded 48 h before harvesting using TriReagent (Sigma-Aldrich, Cat. # T9424). After cDNA synthesis, real-time PCR was performed on an ABI7500 real time PCR machine (Life Technologies, CA). The components and settings of quantitative PCR were determined according to the manual of SYBR Green PCR Master Mix (TAKARA, Cat. # RR820). The primers for amplifying Rad51B, Rad51C, Rad51D, Rad52 and GAPDH were as follows: Rad51B, 5'CCCAAAGATGCAAACGGCTT3' and 5'TCGTCCAAAGCAGAAAGGGT3'; Rad51C, 5' GGATTTGGTGAGTTTCCCGC3' and 5' TCTTTGCTAAGCTCGGAGGG3'; Rad51D, 5' TCTGGCCAAATCTTCCCGAC3' and 5' TCCCAAACAACAGCACAGGT3'; Rad52, 5' CGTTTGCCACCAGAAACCAC3' and 5' TTCCTGTTGTGCGTTGGTCA3'; GAPDH, 5' TGGTATGACAACGAATTTGG3' and 5' TCTACATGGCAACTGTGAGG3'.

### Western blot

Rapidly growing cells were harvested for protein extraction. Thirty μg of each sample was analyzed by Western blot with Rad51C antibody (Abcam, Cat. #, ab72063).

### Transfections and FACS analysis

For examining the promoter activity, all cells were transfected using a Lonza 4D electroporation machine (Lonza, Germany) with according programs: HCA2, IMR90 and WI38, DT130; HMEC1, HMEC2, HMEC3 and HMEC4, EL110; HT1080, FF113; GP2-293 and MCF-7, CM130; Hela, CN114; HCC1954 and T47D, FF150; MDA-MB-231, FF138. Cells were cultured for 72h and harvested for FACS analysis on a FACScalibur (BD Biosciences, USA). Data was further analyzed using Flowjo software. For analyzing the survival rate and inhibitory effect on luciferase activity, cells were transfected using Fugene reagent (Promega, Cat. # E2691).

### Luciferase assay

At 72 h post transfections, cells were harvested and counted before being lysed with passive lysis buffer (Promega, Cat. # E1491) at the ratio of 200 μL/1×10^6^ cells. Then 20 μl of cell lysates was mixed with 100 μl of luciferase substrate (Promega, Cat. # E1910), and the mixture was immediately analyzed on a GloMax20/20 luminometer (Promega, USA).

## SUPPLEMENTARY FIGURES


