# Supplementary material for: Utilization of Rad51C promoter for transcriptional targeting of cancer cells
Source: Oncotarget. 2014 Feb 19;5(7):1805–11. doi: 10.18632/oncotarget.1792 (PMC4039114; doi:10.18632/oncotarget.1792)
Supplement: Supplementary file 1 [file oncotarget-05-1805-s001.pdf]

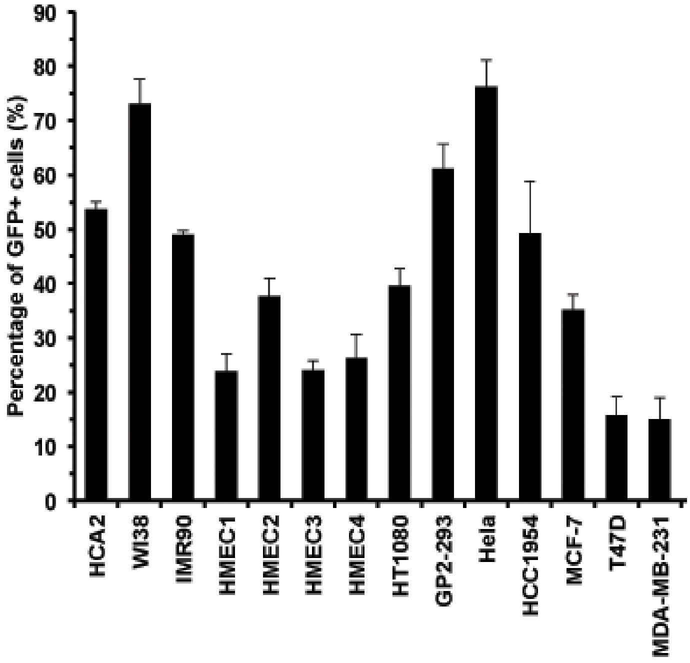

Supplementary Figure 1. Transfection efficiency for each cell line. Cells were transfected with 2 μg pEGFP-N1 using Lonza 4D with different programs. Three days later, cells were harvested for FACS analysis. All experiments were repeated more than three times. Error bars represent s.d.

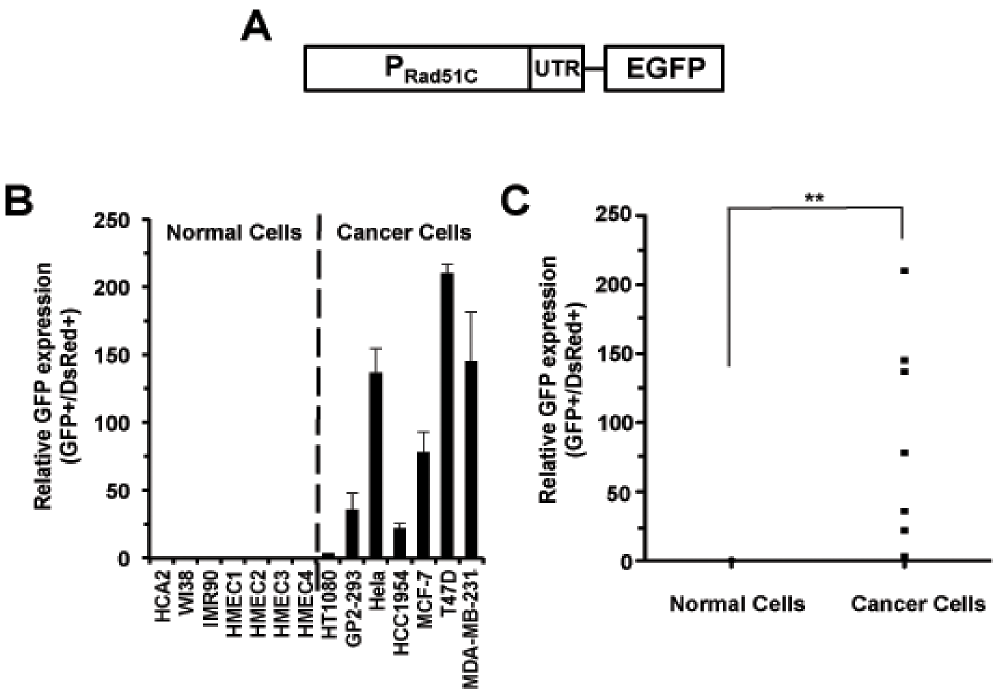

Supplementary Figure 2. The expression of EGFP driven by Rad51C promoter is significantly elevated in cancer cells. **A.** Diagram of Rad51C reporter construct. **B.** The activity of Rad51C promoter is strongly enhanced in cancer cells. Cells were co-transfected with pRad51C-EGFP and pDsRed2-N1. The ratio of GFP+ cells to DsRed+ cells was used as the measure of Rad51C promoter activity. All experiments were repeated more than three times. Error bars indicate s.d. **C.** The elevation of EGFP expression was highly significant in cancer cells. ** P_{MWU}=0.0006.
